# Uncontrolled Donation After Circulatory Death Kidney Transplantation: A Single-Center Experience in Israel with Propensity-Matched Analysis

**DOI:** 10.3390/jcm14228068

**Published:** 2025-11-14

**Authors:** Fahim Kanani, Yael Ben Avraham, Vladimir Tennak, Wadim Mezebovsky, Michael Gurevich, Sigal Eisner, Aviad Gravetz, Eviatar Nesher

**Affiliations:** Department of Transplantation, Beilinson Medical Center, Gray Faculty of Medicine, Tel Aviv University, Petah Tikva 4941492, Israel; 93yaelba@gmail.com (Y.B.A.); vladimirta@clalit.org.il (V.T.); vadymme@clalit.org.il (W.M.); mgurevich@clalit.org.il (M.G.); sigali@clalit.org.il (S.E.); aviadgr@clalit.org.il (A.G.); eviatarne@clalit.org.il (E.N.)

**Keywords:** kidney transplantation, uncontrolled donation after circulatory death, brain death donors, propensity score matching, delayed graft function, Israel

## Abstract

**Background:** Uncontrolled donation after circulatory death (uDCD) remains underutilized globally, despite critical organ shortages. We report outcomes from Israel’s uDCD kidney transplant program compared with the matched donation after brain death (DBD) recipients. **Methods:** This retrospective cohort study analyzed all uDCD kidney transplants performed at the Rabin Medical Center between January 2018 and December 2024, compared with DBD transplants during the same period. Propensity score matching (1:3 ratio) was performed using recipient demographics, comorbidities, and donor characteristics. Primary outcomes included delayed graft function (DGF), graft failure, and patient survival. **Results:** Among 92 kidney transplants, 21 (22.8%) were from uDCD donors. After propensity-matching (21 uDCD, 63 DBD), significant baseline differences persisted: uDCD recipients were younger (47.2 ± 11.8 vs. 57.5 ± 10.9 years, *p* < 0.001) despite a similar dialysis vintage (7.2 ± 3.2 vs. 7.7 ± 3.7 years, *p* = 0.569). Warm ischemia time was 58.5 ± 12.3 vs. 3.0 ± 0.0 min (*p* < 0.001), and cold ischemia time was longer in uDCD (13.7 ± 5.9 vs. 8.4 ± 2.5 h, *p* < 0.001). DGF occurred in 90.5% of uDCD versus 54.1% of DBD recipients (*p* = 0.006). Graft failure was markedly higher in uDCD (28.6% vs. 1.6%, *p* = 0.001), yet mortality was lower (14.3% vs. 27.9%, *p* = 0.339). After a median follow-up of 60 months (IQR 48–72) for both groups, the death-censored 5 year graft survival rate was 71.4% for uDCD versus 98.4% for DBD (*p* < 0.001). **Conclusions:** Despite higher rates of DGF and graft failure, uDCD kidney transplantation demonstrated an acceptable 5 year patient survival rate in carefully selected younger recipients. These findings support cautious expansion of uDCD programs with rigorous recipient selection criteria and realistic outcome expectations.

## 1. Introduction

The global kidney transplant waiting list continues to expand while organ availability remains insufficient, with over 90,000 patients awaiting transplantation in the United States alone, and the median wait time exceeding 4 years [1,2]. This critical shortage has prompted exploration of alternative donor sources beyond traditional donation after brain death (DBD), with donation after circulatory death (DCD) representing the most promising expansion of the donor pool [3]. Among DCD categories, uncontrolled DCD (uDCD) following unexpected cardiac arrest represents a significant but underutilized donor pool. The Maastricht classification distinguishes uDCD (Categories I and II) from controlled DCD (Category III), based on the circumstances of death and timing of organ retrieval [4]. While controlled DCD has gained widespread acceptance with outcomes approaching those of DBD [5], uDCD implementation remains limited to select countries due to complex logistical requirements, ethical considerations, and concerns about inferior outcomes [6,7].

Several European countries have successfully implemented uDCD programs [8,9,10], with Spain reporting that uDCD contributes up to 40% of their deceased donor transplants [11]. France has demonstrated improving outcomes through protocol optimization [12,13], while the Netherlands has shown comparable long-term graft survival rates between uDCD and DBD [14,15]. However, adoption remains limited globally, due to concerns about high rates of delayed graft function (50–90%) and primary non-function compared to DBD transplants [16,17].

Despite growing international experience, critical knowledge gaps persist regarding optimal recipient selection and long-term outcomes. Most published series report only short-term results, and no studies have performed propensity-matched comparisons with extended follow-up [18,19]. The heterogeneity in reported outcomes suggests that program-specific factors and recipient selection significantly influence results [20]. Following the implementation of Israeli Ministry of Health guidelines for DCD in February 2014, our center initiated a uDCD program, emphasizing careful recipient selection. This study aims to compare 5 year outcomes between uDCD and DBD kidney transplants using propensity-matched analysis, and identify factors associated with successful uDCD transplantation. We hypothesized that rigorous recipient selection could achieve comparable long-term patient survival rates, despite the inherent physiological disadvantages of uDCD organs [21].

## 2. Methods

### 2.1. Study Design and Setting

We conducted a retrospective observational cohort study at the Rabin Medical Center, Beilinson Campus, a 1300-bed tertiary care facility serving as the primary transplant center for central and northern Israel. The study period encompassed 1 January 2018 through to 31 December 2024, capturing all consecutive kidney transplants since the inception of our uncontrolled DCD (uDCD) program. The institutional review board approved the protocol (RMC-0804-23, approved 5 December 2023, extended 21 January 2025) with a waiver for informed consent, given the retrospective design.

### 2.2. Regulatory Framework

Israel’s uDCD program operates under guidelines established by the Ministry of Health in February 2014, initially restricting implementation to specialized centers with dedicated rapid response teams. Our institution was among the first authorized centers, developing protocols that balance national guidelines with local capabilities and religious–cultural sensitivities.

### 2.3. uDCD Protocol and Logistics

The uDCD pathway activates for out-of-hospital cardiac arrests in patients aged 10–65 years with witnessed collapse and either bystander CPR or emergency medical services arrival within 15 min. Exclusion criteria include obvious contraindications, such as trauma or known malignancy, and transport time must allow total warm ischemia to be under 150 min. Emergency medical services notify the transplant coordinator simultaneously with hospital notification, allowing the coordinator to activate the procurement team and begin recipient identification while the patient is en route.

In the emergency department, the primary team continues advanced cardiac life support according to standard protocols, while the transplant team remains completely separate. The decision to cease resuscitation is made independently by emergency physicians. Following cessation of resuscitation, a 5 min observation period is observed before death is certified by the emergency physician, using cardiorespiratory criteria, with precise time documentation for warm ischemia calculation.

After death notification, a trained coordinator approaches the family to discuss the uDCD option and explain the process. Written consent is obtained, exceeding legal requirements given historical sensitivities. Preservation is initiated within 30 min of death declaration through mechanical chest compressions using a LUCAS device, endotracheal intubation with 100% oxygen, peripheral IV access for heparin administration (300 units/kg), and femoral cut-down for arterial (15–17 Fr) and venous (21–23 Fr) cannulation, with rapid infusion of cold Ringer’s lactate via femoral lines.

Extracorporeal membrane oxygenation (ECMO) is then initiated using a centrifugal pump with membrane oxygenator, maintaining the target flow at 2.0–2.5 L/min/m^2^, with temperature maintained at 34–36 °C, and continuous pressure monitoring. The patient is transported to the operating room while maintaining ECMO support, with simultaneous recipient preparation.

### 2.4. Surgical Procurement Technique

Surgical procurement begins with midline laparotomy from xiphoid to pubis, immediate aortic control at the diaphragmatic hiatus, and placement of a cross-clamp above the celiac axis, excluding the liver and intestinal circulation. In situ perfusion is performed through aortic cannulation below the renal arteries with the initiation of cold (4 °C) University of Wisconsin solution at a target perfusion pressure of 60–80 mmHg, venous venting via inferior vena cava, and surface cooling with slush ice.

Kidney recovery involves en-bloc mobilization of kidneys with perirenal fat, preservation of maximal ureteral length, careful identification of accessory vessels, and division of renal vessels with adequate patches. Back-table preparation includes separation of the kidneys if procured en-bloc, arterial patch preparation, gentle flushing to clear residual blood, and assessment of the perfusion quality.

### 2.5. Ex Vivo Preservation

All uDCD kidneys undergo mandatory hypothermic machine perfusion, using a LifePort^®^ Kidney Transporter (Organ Recovery Systems, Itasca, IL, USA) with Belzer-MPS^®^ (Bridge to Life Ltd., London, UK) or KPS-1^®^ perfusion solution (Organ Recovery Systems, Itasca, IL, USA). Target parameters include a flow greater than 80 mL/min and resistance less than 0.30 mmHg/mL/min, with continuous monitoring and rejection criteria for resistance exceeding 0.40 or a flow below 60 mL/min.

### 2.6. DBD Comparison Group

DBD organ procurement follows standard protocols with brain death declaration per the 2008 Brain-Respiratory Death Act, donor optimization in the ICU setting, and controlled procurement without warm ischemia. Static cold storage or machine perfusion is selected per surgeon preference.

### 2.7. Warm Ischemic Time Definition

Warm ischemic time (WIT) was defined according to the Israeli Ministry of Health guidelines. For uDCD donors, WIT encompassed the total time from the witnessed cardiac arrest to initiation of the normothermic regional perfusion via ECMO, including both no-flow (cardiac arrest to CPR initiation) and low-flow (CPR duration) periods. For DBD donors, WIT was defined as the time from aortic cross-clamp to the initiation of cold perfusion.

### 2.8. Immunosuppression Protocol

All recipients receive standard center protocol, consisting of induction with basiliximab for standard risk recipients or thymoglobulin for high-risk recipients and those with DGF. Maintenance therapy includes tacrolimus, mycophenolate mofetil, and prednisone, with delayed tacrolimus initiation for DGF cases.

### 2.9. Data Collection and Quality Assurance

Two trained research coordinators independently extracted data from electronic medical records (Chameleon^®^ system, (Kanatek Technologies, Montreal, QC, Canada)), the national transplant registry with mandatory reporting, machine perfusion logs, and operating room databases. Discrepancies were resolved by consensus with transplant nephrologist adjudication when needed. Data completeness was assessed quarterly, with less than 5% missing data for primary outcomes.

Collected variables included recipient demographics, comorbidities, dialysis history, and immunological parameters; donor age, cause of death, terminal creatinine, and medical history; preservation parameters, including warm and cold ischemia times and perfusion parameters; perioperative data including surgical complications, immunosuppression, and length of stay; and outcomes including graft function, rejection episodes, and patient and graft survival rates.

### 2.10. Outcome Definitions

Primary outcomes included delayed graft function (DGF), defined as a need for dialysis within the first 7 days post-transplant. Unlike historical definitions requiring multiple sessions or excluding single sessions for hyperkalemia, we adopted the current consensus definition, wherein any dialysis constitutes DGF, reflecting contemporary practice. Graft failure was defined as a composite endpoint, including a permanent return to dialysis beyond 90 days, re-transplantation, or death with a functioning graft (included per regulatory requirements). Patient mortality included all-cause death, which was censored at the last follow-up.

Secondary outcomes comprised primary non-function (PNF), defined as graft producing less than 200 mL urine per 24 h and requiring continuous dialysis from transplant; functional DGF, defined as urine output exceeding 500 mL per 24 h but still requiring dialysis; serum creatinine trajectory, with protocol measurements at discharge, 1, 3, 6, and 12 months, then annually; estimated GFR, calculated using the CKD-EPI equation; acute rejection, either biopsy-proven according to Banff [22] criteria or clinically treated; and surgical complications, classified according to the Clavien–Dindo classification. Causes of death and graft failure were systematically recorded and classified.

The follow-up duration was calculated from the transplant date to the last clinical visit, death, or 31 December 2024. All patients had a minimum of a 5 year follow-up, with serum creatinine measurements available at all protocol time points.

### 2.11. Statistical Analysis

To address inherent selection bias in uDCD allocation, we employed propensity score matching. Propensity scores were estimated using logistic regression, with uDCD as the outcome variable, including covariates selected a priori, based on clinical relevance: age, sex, BMI, diabetes, hypertension, coronary disease, dialysis vintage, dialysis modality, smoking history, terminal donor creatinine, and ABO blood type. Matching was performed using a nearest-neighbor algorithm without replacement at a 1:3 ratio (uDCD:DBD), with a caliper width of 0.2 standard deviations of the propensity score logit. Balance was assessed using standardized mean differences (SMD), with values exceeding 0.2 indicating a meaningful imbalance.

The analytical approach included descriptive statistics with mean ± SD for normally distributed data and the median, with the interquartile range for skewed data. Bivariate comparisons used Student’s t-test or Mann–Whitney U test for continuous variables and chi-square or Fisher’s exact test for categorical variables. Time-to-event analysis employed Kaplan–Meier curves with log-rank tests.

Multivariable adjustment utilized a simplified Cox proportional hazards regression model including only donor type (uDCD versus DBD) and recipient age, given our limited sample size of 21 uDCD cases with fewer than 20 total mortality events. This approach follows the rule of thumb of 10 events per variable, to ensure model stability. Variables for adjustment were selected based on clinical importance and univariate *p* < 0.10, with the proportional hazard assumption being verified using Schoenfeld residuals.

Longitudinal analysis used linear mixed-effects models for the creatinine trajectory, with random intercepts for patients and time being treated as a fixed effect with group × time interaction.

Sensitivity analyses included alternative matching ratios (1:2, 1:4), inclusion of perfusion parameters for outcome models, competing risk analysis for graft failure with death as a competing event, and multiple imputation for missing baseline laboratories.

All analyses were performed using R version 4.3.1 (R Foundation, Vienna, Austria), with packages MatchIt (v4.5.0), survival (v3.5-5), lme4 (v1.1-33), and tidyverse (v2.0.0). Statistical code is available upon request. Statistical significance was defined as two-tailed *p* < 0.05 with no adjustment for multiple comparisons, given the exploratory nature of secondary analyses.

## 3. Results

### 3.1. Study Population Characteristics

During the 7 year study period, 92 kidney transplants met the inclusion criteria: 21 (22.8%) from uDCD donors and 71 (77.2%) from DBD donors. The annual number of uDCD transplants increased from two in 2018 to five in 2024.

Before matching, uDCD recipients were younger than DBD recipients (47.2 ± 11.8 vs. 58.6 ± 10.8 years, *p* < 0.001), with a similar dialysis vintage (7.2 ± 3.2 vs. 7.9 ± 3.8 years, *p* = 0.440). uDCD recipients had lower rates of diabetes mellitus (19.0% vs. 47.1%, *p* = 0.042) and higher hypertension prevalence (71.4% vs. 39.4%, *p* = 0.020) (Table 1).

### 3.2. Propensity Score Matching Results

Propensity achieved a 1:3 ratio, with all 21 uDCD recipients matched to 63 DBD recipients. After matching, the SMD decreased for BMI (0.118 to 0.073), dialysis duration (0.203 to 0.150), and smoking history (0.112 to 0.176). SMDs remained >0.2 for age (0.905), diabetes (0.472), and hypertension (0.568) (Table 2).

### 3.3. Donor and Procurement Characteristics

Donor cause of death differed between groups (*p* < 0.001, SMD = 2.685): cardiac causes in 76.2% of uDCD vs. 0% of DBD donors; cerebrovascular accidents in 4.8% of uDCD vs. 54.0% of DBD donors. Terminal donor creatinine was 1.9 ± 0.1 mg/dL in uDCD vs. 1.2 ± 1.3 mg/dL in DBD donors (*p* = 0.042). Cold ischemia time was 13.7 ± 5.9 h in uDCD vs. 8.4 ± 2.5 h in DBD (*p* < 0.001, SMD = 1.164). Warm ischemia time was 58.5 ± 12.3 min in uDCD vs. 3.0 ± 0.0 min in DBD (*p* < 0.001) (Table 3).

### 3.4. Clinical Outcomes

**Figure 1:**
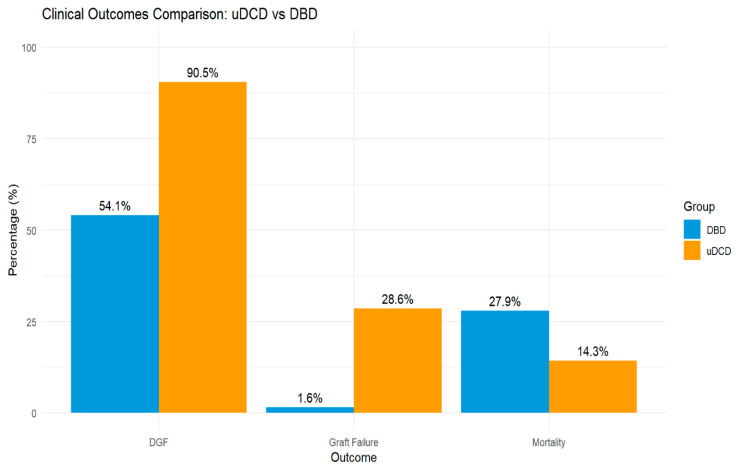
Clinical outcomes comparison between uDCD and DBD kidney transplant recipients after propensity score matching. DGF = delayed graft function.

DGF occurred in 19/21 (90.5%) uDCD recipients vs. 33/61 (54.1%) DBD recipients (*p* = 0.006, SMD = 0.890). Primary non-function occurred in 2/21 (9.5%) uDCD vs. 1/63 (1.6%) DBD recipients (*p* = 0.238). Graft failure occurred in 6/21 (28.6%) uDCD vs. 1/63 (1.6%) DBD recipients (*p* = 0.001). Patient mortality was 3/21 (14.3%) in uDCD vs. 17/61 (27.9%) in DBD recipients (*p* = 0.339). After the median follow-up of 60 months (IQR 48–72) for both groups, the death-censored graft survival rate at 5 years was 71.4% for uDCD and 98.4% for DBD (log-rank *p* < 0.001) (Figure 1).

#### 3.4.1. Causes of Death and Graft Failure

Among 20 deaths with available cause data, cardiovascular causes accounted for 11/20 (55%) and infection/other causes for 9/20 (45%). All deaths occurred with functioning grafts. The six graft failures in uDCD recipients occurred at a median of 18 months (range 3–48), due to chronic rejection (n = 3), acute rejection (n = 2), and recurrent disease (n = 1). The single DBD graft failure occurred at 36 months, due to chronic rejection.

#### 3.4.2. Survival Analysis

**Figure 2:**
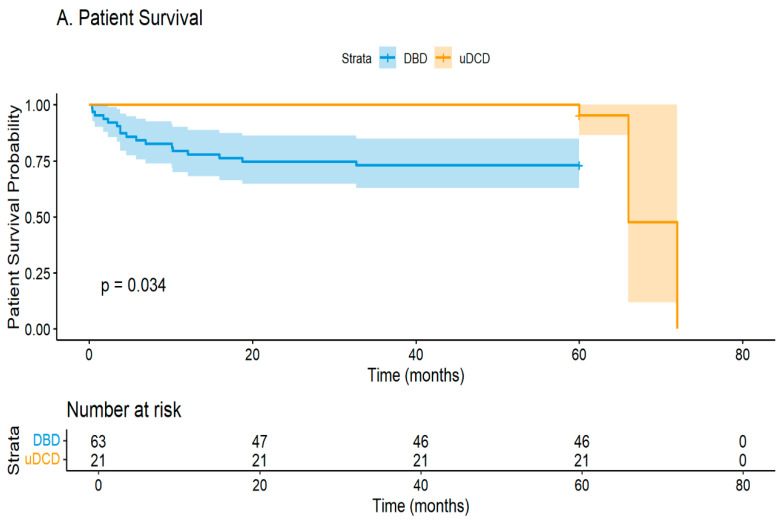

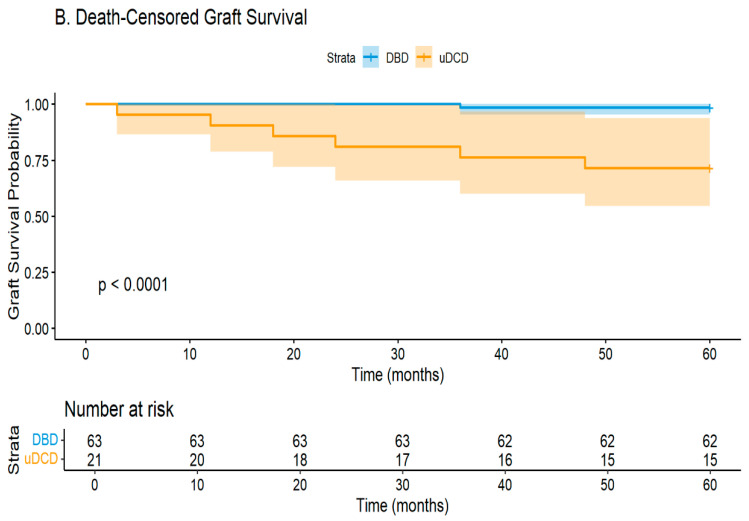
Kaplan–Meier curves for (**A**) patient survival and (**B**) death-censored graft survival. Numbers at risk shown below each curve.

Kaplan–Meier analysis revealed significant differences in the graft survival rate between groups. The death-censored 5 year graft survival rate was 71.4% for uDCD versus 98.4% for DBD recipients (log-rank *p* < 0.001) (Figure 2B). Patient survival showed a non-significant trend favoring uDCD recipients, with a 5 year survival rate of 85.7% versus 72.1% (log-rank *p* = 0.209) (Figure 2A).

Cox proportional hazards regression was performed with limited covariates due to sample size constraints. In univariate analysis, uDCD status showed a protective association with mortality (HR 0.092, 95% CI 0.011–0.729, *p* = 0.024). However, after adjustment for recipient age, this association was attenuated (HR 0.194, 95% CI 0.013–2.87, *p* = 0.233). Recipient age remained the only significant predictor of mortality in the multivariate model (HR 1.16 per year increase, 95% CI 1.01–1.35, *p* = 0.041) (Table 4).

### 3.5. Renal Function Trajectory

**Figure 3:**
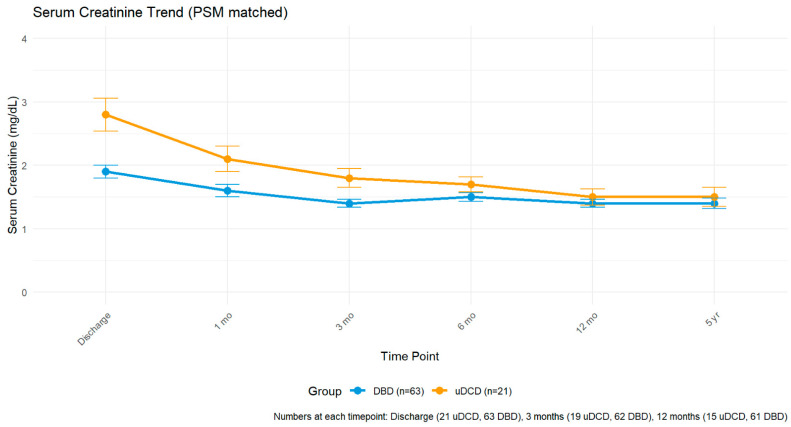
Mean serum creatinine trajectory among patients with functioning grafts. Error bars represent standard error. Numbers at each time point: discharge (21 uDCD, 63 DBD), 3 months (19 uDCD, 62 DBD), 12 months (15 uDCD, 61 DBD). Y-axis: serum creatinine (mg/dL).

Mean was 2.8 ± 1.2 mg/dL in uDCD vs. 1.9 ± 0.8 mg/dL in DBD (*p* = 0.002). At 3 months: 1.8 ± 0.7 vs. 1.4 ± 0.5 mg/dL (*p* = 0.021). At 12 months among functioning grafts: 1.5 ± 0.6 vs. 1.4 ± 0.5 mg/dL (*p* = 0.486) (Figure 3).

## 4. Discussion

Our seven year experience with uncontrolled DCD kidney transplantation reveals encouraging results that support cautious program expansion. Despite a ninety percent delayed graft function rate, nineteen of twenty-one kidneys (90.5%) achieved function within a median of eighteen days, with only two cases (9.5%) of primary non-function requiring permanent dialysis. Notably, six grafts (28.6%) failed during our median five year follow-up, primarily due to rejection, while the patient survival rate remained acceptable at 85.7% in our carefully selected younger cohort.

### 4.1. Contextualizing Our Outcomes Within International Experience

The ninety percent DGF rate observed in our uDCD cohort substantially exceeds most international benchmarks, yet aligns with recent reports from centers using similar protocols. The systematic review by Rijkse and colleagues reported DGF rates of approximately fifty to sixty percent across mixed DCD populations, though these included predominantly controlled DCD with inherently better expected outcomes [1]. More relevant comparisons come from uDCD-specific series: Pinho and colleagues in Portugal reported seventy percent DGF, despite using normothermic regional perfusion [21], while the meta-analysis by Vijayan found sixty-five percent DGF rates specifically in uDCD cohorts [10].

Our mean warm ischemic time of 58.5 ± 12.3 min falls within the expected range for uDCD programs internationally, where 40–80 min is typical [23]. This duration, encompassing both no-flow and low-flow periods according to Israeli Ministry of Health protocols, likely contributes to our high DGF rate but remains within acceptable limits that permit organ recovery.

Critically, our high DGF rate must be interpreted alongside the crucial finding that ninety percent of these kidneys ultimately functioned. The median time to function of eighteen days, while prolonged, demonstrates that patience during the recovery period yields acceptable outcomes. This contrasts with early uDCD experiences, where high DGF often presaged permanent non-function.

### 4.2. Understanding Age Allocation and Selection Effects

The eleven year age gap between cohorts reflects Israel’s organ allocation system, which prioritizes donor–recipient age matching. Younger recipients thus have greater access to uDCD organs, which typically come from younger donors following cardiac arrest. This allocation principle, while creating statistical imbalance that propensity-matching cannot fully address, may paradoxically benefit outcomes—younger recipients possess a greater physiological reserve to tolerate the ischemic insult that is inherent to uDCD procurement.

The mortality paradox in our study—14.3% in uDCD versus 27.9% in DBD recipients—largely reflects this age difference. Our analysis of causes of death revealed that all deaths occurred with functioning grafts, with cardiovascular causes predominating (55%), followed by infections (45%). The higher mortality in older DBD recipients aligns with expected age-related mortality, rather than transplant-specific factors.

The similar dialysis vintage between groups (7.2 versus 7.7 years), despite the age difference, provides reassurance that uDCD organs are not being allocated to desperate cases with prolonged waiting times. Rather, the allocation reflects systematic age-matching principles that may optimize long-term outcomes.

A particularly troubling finding in our cohort was the markedly higher graft loss from rejection in uDCD recipients. Among the six graft failures (28.6%), five were rejection-related: three from chronic rejection and two from acute rejection. This contrasts starkly with the single graft failure (1.6%) in the DBD group, which was also due to chronic rejection. This six-fold increase in rejection-mediated graft loss demands careful consideration and has direct implications for future recipient selection and management protocols.

Several factors may contribute to this elevated rejection risk. First, the severe ischemia–reperfusion injury that is inherent to uDCD procurement triggers a robust inflammatory cascade that may prime the alloimmune response. The prolonged warm ischemia (58.5 ± 12.3 min), followed by mandatory machine perfusion, creates a ‘danger signal’ milieu that enhances antigen presentation and T-cell activation. Second, the extended DGF period (90.5% incidence) delays therapeutic immunosuppression optimization, as tacrolimus initiation is typically postponed during dialysis-dependent recovery. This immunosuppressive lag during a critical inflammatory period may permit subclinical rejection episodes that manifest later as a chronic allograft injury.

These findings suggest that current immunosuppression protocols, developed primarily for DBD transplants, may be inadequate for the unique immunological challenges of uDCD organs. Future management strategies should consider the following: (1) enhanced induction therapy, potentially with alemtuzumab rather than basiliximab, even in low-immunological risk recipients; (2) earlier initiation of calcineurin inhibitors with careful therapeutic monitoring during the DGF period; (3) protocol biopsies at 3 and 12 months to detect subclinical rejection; and (4) biomarker surveillance for early rejection detection.

Prospective studies comparing intensified immunosuppression protocols in uDCD recipients are urgently needed to address this critical limitation of current practice.

### 4.3. The Israeli Context: Historical Legacy and Contemporary Practice

Our experience cannot be fully understood without acknowledging the profound impact of the 1993 Soroka Medical Center incident, which fundamentally altered Israeli organ procurement practice. Despite the Law of Anatomy and Pathology technically permitting organ procurement without explicit consent, this traumatic event established a de facto expressed consent policy, requiring family agreement [18]. This historical context creates unique challenges for uDCD implementation, where rapid decision-making is essential yet must navigate heightened sensitivities around consent and bodily autonomy.

The February 2014 Ministry of Health guidelines permitting uDCD represented more than regulatory evolution—they embodied a delicate negotiation between expanding organ availability and maintaining public trust. With only four percent of Israelis holding Adi donor cards and fewer than three hundred organs available annually for over eight hundred waiting patients [18], the pressure for alternative donation pathways was immense. Yet, these guidelines emerged against a backdrop of historical controversy, requiring stringent safeguards: witnessed arrests, CPR initiation within fifteen minutes, and warm ischemia capped at one hundred and fifty minutes.

### 4.4. The Religious–Cultural Paradox

Perhaps nowhere is the complexity of Israeli organ donation more apparent than in the religious dimension. While mainstream Halakhic interpretation, formally adopted by the Israeli Rabbinate in 1982, considers organ donation for life-saving purposes to be a mitzvah (moral obligation), approximately forty-five percent of families who refuse donation cite religious concerns [18]. This paradox extends deeper: as Rabbi Shaul Israeli noted, Halakhic principles actually align more closely with presumed consent than expressed consent—in the absence of explicit objection, organs should be procured for life-saving purposes, with family objection lacking validity under Jewish law [18].

For uDCD programs, where cardiac death is unambiguous compared to the Halakhic debates surrounding brain death, one might expect greater religious acceptance. However, our experience suggests that cultural anxieties about death determination and bodily integrity transcend specific definitions of death, requiring continued sensitivity in family approaches.

### 4.5. Technical Factors and Protocol Considerations

The 13.7 h mean cold ischemia time warrants clarification. This duration reflects our mandatory protocol, requiring at least twelve hours of hypothermic machine perfusion to assess organ viability through resistance parameters. Importantly, no organs demonstrated prohibitive resistance requiring discard, validating this conservative approach. While this extends cold ischemia beyond the international targets of eight hours, the protocol serves dual purposes: optimizing organ selection and building program confidence during the learning-curve phase.

The inclusion of terminal donor creatinine in our analysis (1.9 ± 0.1 mg/dL for uDCD versus 1.2 ± 1.3 mg/dL for DBD, *p* = 0.042) highlights another selection factor. The higher terminal creatinine in uDCD donors likely reflects the physiological stress of cardiac arrest and resuscitation attempts, yet these organs achieved acceptable function, suggesting that mild elevation in terminal creatinine should not preclude uDCD organ utilization [24].

All uDCD kidneys underwent hypothermic machine perfusion, following evidence-based guidelines. The universal achievement of acceptable perfusion parameters despite prolonged warm ischemia suggests that machine perfusion effectively mitigates ischemic injury in carefully selected organs.

### 4.6. Comparative Context Within Our Center

Our institution’s broader transplant outcomes provide essential context for interpreting uDCD results. With over one hundred and fifty living donor transplants and fifty DBD transplants annually, we maintain greater than ninety percent five year graft and patient survival rates. Against this benchmark of excellence, the uDCD outcomes—while showing higher graft failure (28.6% versus 1.6%) and DGF rates—remain acceptable, particularly given the 71.4% death-censored five year graft survival rate.

This comparison highlights a critical consideration: patients choosing between waiting for living donors versus accepting uDCD organs face complex trade-offs. While living donation offers superior outcomes, the immediate availability of uDCD organs may benefit younger patients accumulating dialysis-related morbidity during extended wait times.

### 4.7. Resource Utilization and Ethical Considerations

The economic implications of our uDCD program appear favorable, despite high DGF rates. With only 9.5% primary non-function and 71.4% five year graft survival rates, over seventy percent of recipients avoid returning to chronic dialysis long-term. Given the substantial costs of dialysis—both economic and quality-of-life—successful uDCD transplantation represents efficient resource utilization, even accounting for extended initial hospitalization during DGF recovery [25,26].

Our informed consent process has evolved to address the unique challenges of uDCD allocation. We explicitly discuss the ninety percent likelihood of requiring temporary dialysis post-transplant, while emphasizing that nineteen of twenty-one kidneys in our experience achieved function within three weeks. We also discuss the higher risk of graft failure compared to DBD transplants. This balanced presentation allows patients to make informed decisions, particularly younger patients who may benefit from accepting a higher short-term risk for long-term dialysis freedom.

### 4.8. Future Directions

#### 4.8.1. The Promise of Normothermic Perfusion

Looking forward, normothermic regional perfusion (NRP) represents a promising technology that could substantially improve our outcomes. International experience suggests that NRP reduces DGF rates and improves early function by restoring cellular metabolism before organ recovery. Implementation in Israel faces regulatory, resource, and potentially religious barriers, requiring careful navigation. However, given our demonstration of program safety with the current protocols, evolution toward NRP merits serious consideration.

#### 4.8.2. The Need for KDPI Implementation in Israel

Our findings highlight a critical gap in Israeli transplant practice—the absence of a validated risk stratification system such as the kidney donor profile index (KDPI). The markedly elevated graft loss rate among uDCD recipients (28.6% vs. 1.6% in DBD) underscores the urgent need for more nuanced donor–recipient matching, beyond our current age-based allocation system.

The KDPI, which incorporates ten donor characteristics including age, creatinine, hypertension, diabetes, cause of death, and DCD status, has proven valuable internationally for predicting graft outcomes. Recent validation studies in Chinese populations have demonstrated that while KDPI > 85% kidneys show inferior long-term function compared to KDPI ≤ 85% organs, they maintain acceptable graft and patient survival rates when appropriately matched to recipients. Shui et al. reported that KDPI > 85% kidneys achieved an 88.1% four year graft survival rate, despite significantly worse renal function parameters, supporting their utility in carefully selected recipients.

Implementation of KDPI scoring in Israel would enable the following:

1. More informed consent discussions with recipients about expected outcomes.
2. Optimization of donor–recipient matching beyond simple age criteria.
3. Objective comparison of uDCD organs with other marginal donors.
4. Risk-adjusted outcome, reporting to benchmark program performance.

Our current practice of preferentially allocating uDCD organs to younger recipients may paradoxically increase immunological risk, as suggested by our high rejection rates. A KDPI-based system could identify older recipients with a lower immunological risk, who might achieve superior outcomes despite accepting organs with higher KDPI scores. This approach has proven successful internationally, where recipients over 60 years show equivalent survival rates when transplanted with KDPI > 85% versus standard criteria organs.

Until formal KDPI implementation, we propose developing an interim risk score, incorporating locally relevant factors such as warm ischemia time, machine perfusion parameters, and recipient age to guide allocation decisions and improve transparency in the consent process [27].

### 4.9. Study Limitations

Our study’s limitations include the small sample size limiting statistical power, single-center design potentially limiting generalizability, and different follow-up durations introducing temporal biases. Unmeasured confounding certainly exists—the clinical decision-making underlying recipient selection for uDCD allocation was not captured, but undoubtedly influences outcomes.

Second, substantial selection bias persists despite rigorous propensity score matching. The inability to balance age (SMD = 0.905), diabetes (SMD = 0.472), and other key variables reflects the fundamental differences in populations, rather than a methodological failure. Multiple analytical approaches, including alternative matching ratios, failed to achieve adequate balance without excessive case loss.

Third, a single-center design from one of Israel’s pioneering uDCD programs limits generalizability. Our outcomes likely reflect learning curve effects, evolving protocols, and institution-specific factors that may not translate to centers with different resources or experience.

Fourth, unmeasured confounding significantly impacts results interpretation. Critical factors influencing allocation decisions—clinical judgment, family dynamics, and real-time organ quality assessment—remain uncaptured in administrative databases. These hidden variables may explain the substantial outcome variation.

Fifth, Israel’s unique religious–cultural context creates specific selection pressures. The intersection of Halakhic interpretation, cultural attitudes toward brain death, and historical incidents shapes both donor availability and recipient selection in ways that are absent from secular healthcare systems.

Sixth, a critical methodological limitation involves the differential use of machine perfusion between groups. All uDCD kidneys underwent mandatory hypothermic machine perfusion for at least 12 h, while DBD kidneys received either static cold storage or machine perfusion per surgeon preference. This systematic difference in preservation methods represents an unmeasured confounder that may have influenced outcomes, particularly the observed differences in DGF rates. The protective effect of machine perfusion on ischemic injury is well-established, potentially mitigating some adverse effects of prolonged warm ischemia in uDCD organs. This preservation bias should be considered when interpreting the comparative outcomes and limits the validity of our propensity-matching, which could not account for this systematic protocol difference.

## 5. Conclusions

Our single-center experience demonstrates that uncontrolled DCD kidney transplantation, while associated with high rates of delayed graft function and increased graft failure risk, provides acceptable five year outcomes for carefully selected younger recipients. The ninety percent ultimate function rate, 71.4% death-censored five year graft survival rate, and acceptable patient survival support continued program development. Success requires acknowledging that uDCD kidneys demand patience during recovery, meticulous preservation protocols, and transparent communication about expected outcomes. Within Israel’s unique religious–cultural context and age-based allocation system, uDCD offers a valuable pathway to expand organ availability while maintaining public trust through careful protocol adherence.

## Figures and Tables

**Table 1:** Baseline characteristics before propensity score matching.

Characteristics	uDCD (n = 21)	DBD (n = 71)	*p*-Value	SMD
Recipient Demographics				
Age, years	47.2 ± 11.8	58.6 ± 10.8	<0.001	1.003
Male sex, n (%)	15 (71.4)	42 (59.2)	0.446	0.260
BMI, kg/m^2^	26.7 ± 4.4	27.3 ± 5.5	0.656	0.118
Obesity (BMI ≥ 30), n (%)	4 (19.0)	26 (36.6)	0.213	0.400
Dialysis Characteristics				
Dialysis duration, years	7.2 ± 3.2	7.9 ± 3.8	0.440	0.203
Dialysis modality, n (%)			0.149	0.616
Hemodialysis	21 (100.0)	58 (84.1)		
Peritoneal dialysis	0 (0.0)	8 (11.6)		
Both	0 (0.0)	3 (4.3)		
Recipient Blood Type, n (%)			0.190	0.739
A−	2 (9.5)	1 (1.5)		
A+	10 (47.6)	24 (35.8)		
AB+	0 (0.0)	4 (6.0)		
B−	1 (4.8)	2 (3.0)		
B+	1 (4.8)	14 (20.9)		
O+	7 (33.3)	22 (32.8)		
Comorbidities, n (%)				
Diabetes mellitus	4 (19.0)	32 (47.1)	0.042	0.624
Hypertension	15 (71.4)	28 (39.4)	0.020	0.680
Ischemic heart disease	3 (14.3)	5 (7.0)	0.552	0.236
Smoking history	10 (47.6)	33 (53.2)	0.848	0.112
Pulmonary disease	2 (9.5)	20 (29.4)	0.119	0.519
Rheumatologic disease	2 (9.5)	16 (23.5)	0.278	0.384
Urologic history	0 (0.0)	7 (10.3)	0.285	0.479
Hypercoagulability	2/2 (100.0)	13/31 (41.9)	0.387	1.664
Serology, n/tested (%)				
CMV positive	3/3 (100.0)	13/15 (86.7)	1.000	0.555
HCV/HBV/HIV positive	0/5 (0.0)	7/44 (15.9)	0.670	0.615

**Table 2:** Characteristics after propensity score matching (1:3 ratio).

Characteristics	uDCD (n = 21)	DBD (n = 63)	*p*-Value	SMD
Recipient Demographics				
Age, years	47.2 ± 11.8	57.5 ± 10.9	<0.001	0.905
Male sex, n (%)	15 (71.4)	38 (60.3)	0.514	0.236
BMI, kg/m^2^	26.7 ± 4.4	27.0 ± 5.5	0.786	0.073
Obesity (BMI ≥ 30), n (%)	4 (19.0)	22 (34.9)	0.276	0.363
Comorbidities, n (%)				
Diabetes mellitus	4 (19.0)	24 (40.0)	0.141	0.472
Hypertension	15 (71.4)	28 (44.4)	0.059	0.568
Ischemic heart disease	3 (14.3)	5 (7.9)	0.668	0.203
Donor Characteristics				
Donor age, years	48.3 ± 9.5	54.4 ± 15.8	0.099	0.468
Donor age > 50 years, n (%)	14 (66.7)	45 (71.4)	0.890	0.103
Male donor, n (%)	18 (85.7)	43 (68.3)	0.204	0.424
Donor Cause of Death, n (%)			<0.001	2.685
Anoxia	2 (9.5)	14 (22.2)		
Cardiac arrest	16 (76.2)	0 (0.0)		
Cerebrovascular accident	1 (4.8)	34 (54.0)		
Trauma	2 (9.5)	15 (23.8)		
Donor Blood Type, n (%)			0.376	0.493
A	12 (57.1)	25 (39.7)		
AB	0 (0.0)	2 (3.2)		
B	2 (9.5)	14 (22.2)		
O	7 (33.3)	22 (34.9)		

**Table 3:** Transplant characteristics and outcomes after matching.

Characteristics	uDCD (n = 21)	DBD (n = 63)	*p*-Value	SMD
Graft Characteristics				
Graft side (left), n (%)	6 (28.6)	25 (39.7)	0.514	0.236
Number of arteries, n (%)			0.501	0.352
Single	18 (85.7)	48 (76.2)		
Two	3 (14.3)	12 (19.0)		
Three	0 (0.0)	3 (4.8)		
Complex venous anatomy, n (%)	0 (0.0)	5 (7.9)	0.424	0.415
Ischemia Times				
Cold ischemia time, hours #	13.7 ± 5.9	8.4 ± 2.5	<0.001	1.164
Warm ischemia time, minutes	58.5 ± 12.3	3.0 ± 0.0	<0.001	-
Terminal donor creatinine, mg/dL	1.9 ± 0.1	1.2 ± 1.3	0.042	-
Surgical Characteristics				
Total operative time, hours *	3.0 ± 0.9	3.3 ± 1.0	0.266	0.288
Reoperation, n (%)	1 (4.8)	1 (1.6)	1.000	0.178
Postoperative bleeding, units	0.3 ± 0.5	0.2 ± 0.4	0.310	0.247
Immunosuppression				
Induction therapy, n (%)			—	—
Basiliximab (Simulect)	—	—		
Thymoglobulin	—	—		
Clinical Outcomes				
Delayed graft function †, n (%)	19 (90.5)	33 (54.1)	0.006	0.890
Biopsy	21	10		
Biopsy Timing	10 (8–12)	15 (12–18)		
Graf functioning (days)	18 (10–30)	3 (1–4)		
Primary non-function, n/evaluated (%)	2 (9.5)	1 (1.6)	0.238	0.296
Graft Failure, n (%)	**6 (28.6)**	1 **(1.6)**	**0.001**	**0.814**
Patient mortality, n (%)	3 (14.3)	17 (27.9)	0.339	0.338
Follow-Up, Months, Median (IQR)	**60 (48–72)**	**60 (48–72)**	**—**	**—**

# All uDCD kidneys were put on a hypothermic machine for at least 12 h. * Operative time was defined as the total duration from skin incision to skin closure in the recipient, excluding donor retrieval and machine perfusion times. † Defined as need for dialysis within first 7 days post-transplant. Total time from incision to closure.

**Table 4:** Simplified Cox proportional hazards model for patient mortality.

Variable	HR (95% CI)	*p*-Value
Donor type (uDCD vs. DBD)	0.19 (0.01–2.87)	0.233
Recipient age (per year)	1.16 (1.01–1.35)	0.041

## Data Availability

The datasets analyzed during the current study are available from the corresponding author upon reasonable request, subject to institutional data-sharing agreements.

## References

[1] Rijkse E., Ceuppens S., Qi H., Ijzermans J.N.M., Hesselink D.A., Minnee R.C. (2021). Implementation of donation after circulatory death kidney transplantation can safely enlarge the donor pool: A systematic review and meta-analysis. Int. J. Surg..

[2] Schaapherder A., Wijermars L.G.M., de Vries D.K., de Vries A.P.J., Bemelman F.J., van de Wetering J., van Zuilen A.D., Christiaans M.H.L., Hilbrands L.H., Baas M.C. (2018). Equivalent long-term transplantation outcomes for kidneys donated after brain death and cardiac death: Conclusions from a nationwide evaluation. EClinicalMedicine.

[3] Summers D.M., Johnson R.J., Allen J., Fuggle S.V., Collett D., Watson C.J., Bradley J.A. (2010). Analysis of factors that affect outcome after transplantation of kidneys donated after cardiac death in the UK: A cohort study. Lancet.

[4] Kootstra G., Daemen J.H., Oomen A.P. (1995). Categories of non-heart-beating donors. Transplant. Proc..

[5] Lomero M., Gardiner D., Coll E., Haase-Kromwijk B., Procaccio F., Immer F., Gabbasova L., Antoine C., Jushinskis J., Lynch N. (2020). Donation after circulatory death today: An updated overview of the European landscape. Transpl. Int..

[6] Oniscu G.C., Randle L.V., Muiesan P., Butler A.J., Currie I.S., Perera M.T., Forsythe J.L., Watson C.J.E. (2014). In situ normothermic regional perfusion for controlled donation after circulatory death--the United Kingdom experience. Am. J. Transplant..

[7] Ortega-Deballon I., Hornby L., Shemie S.D. (2015). Protocols for uncontrolled donation after circulatory death: A systematic review of international guidelines, practices and transplant outcomes. Crit. Care.

[8] Manara A.R., Murphy P.G., O’Callaghan G. (2012). Donation after circulatory death. Br. J. Anaesth..

[9] Hoogland E.R.P., Snoeijs M.G.J., Winkens B., Christaans M.H.L., van Heurn L.W.E. (2011). Kidney transplantation from donors after cardiac death: Uncontrolled versus controlled donation. Am. J. Transplant..

[10] Vijayan K., Schroder H.J., Hameed A., Hitos K., Lo W., Laurence J.M., Yoon P.D., Nahm C., Lim W.H., Lee T. (2024). Kidney transplantation outcomes from uncontrolled donation after circulatory death: A systematic review and meta-analysis. Transplantation.

[11] Hessheimer A.J., Coll E., Torres F., Ruíz P., Gastaca M., Rivas J.I., Gómez M., Sánchez B., Santoyo J., Ramírez P. (2019). Normothermic regional perfusion vs. super-rapid recovery in controlled donation after circulatory death liver transplantation. J. Hepatol..

[12] Hosgood S.A., Saeb-Parsy K., Hamed M.O., Nicholson M.L. (2016). Successful transplantation of human kidneys deemed untransplantable but resuscitated by ex-vivo normothermic machine perfusion. Am. J. Transplant..

[13] Sánchez-Fructuoso A.I., Prats D., Torrente J., Pérez-Contín M.J., Fernández C., Alvarez J., Barrientos A. (2000). Renal transplantation from non-heart beating donors: A promising alternative to enlarge the donor pool. J. Am. Soc. Nephrol..

[14] Antoine C., Savoye E., Gaudez F., Cheisson G., Badet L., Videcoq M., Legeai C., Bastien O., Barrou B. (2020). Kidney transplant from uncontrolled donation after circulatory death: Contribution of normothermic regional perfusion. Transplantation.

[15] Peters-Sengers H., Houtzager J.H.E., Idu M.M., Heemskerk M.B.A., van Heurn E.L.W., van der Heide J.J.H., Kers J., Berger S.P., van Gulik T.M., Bemelman F.J. (2019). Impact of cold ischemia time on outcomes of kidney transplantation from donation after circulatory death donors: A Dutch cohort study. Transplant. Direct.

[16] Jochmans I., Nicholson M.L., Hosgood S.A. (2017). Kidney perfusion: Some like it hot others prefer to keep it cool. Curr. Opin. Organ Transplant..

[17] Hosgood S.A., Thompson E., Moore T., Wilson C.H., Nicholson M.L. (2018). Normothermic machine perfusion for the assessment and transplantation of declined human kidneys from donation after circulatory death donors. Br. J. Surg..

[18] Scott O., Jacobson E. (2007). Implementing presumed consent for organ donation in Israel: Public, religious and ethical issues. IMAJ.

[19] Elmer A., Rohrer M.L., Benden C., Krügel N., Beyeler F., Immer F.F. (2022). Organ donation after circulatory death as compared with organ donation after brain death in Switzerland—an observational study. Swiss Med. Wkly..

[20] Rouhi A.D., Choudhury R.A., Hoeltzel G.D., Prins K., Yoeli D., Moore H.B., Williams N.N., Dumon K.R., Nydam T.L. (2023). Uncontrolled donation after cardiac death kidney transplantation: Opportunity to expand the donor pool?. Am. J. Surg..

[21] Pinho A., Sampaio S., Alencastre I., Polidoro M.J., Rios M., Roncon-Albuquerque R., Silva J., Silva C., Pestana M. (2025). Kidney transplantation from uncontrolled donation after circulatory death maintained by normothermic regional perfusion: An 8-year Portuguese single-centre experience. Transpl. Int..

[22] Banff Foundation for Allograft Pathology Banff Classification of Renal Transplant Pathology, Version 2023-2. https://banfffoundation.org/central-repository-for-banff-classification-resources-3/.

[23] Roman J., Jalůvka F., Ostruszka P., Jelínek P., Hrubovčák J., Havránek P., Vrtková A., Lys Z., Dědochová J., Procházka V. (2022). Post-kidney transplantation results after circulatory or brain death without pre-mortem heparin administration. Med. Sci. Monit..

[24] Cho Y.W., Terasaki P.I., Cecka J.M., Gjertson D.W. (1998). Transplantation of kidneys from donors whose hearts have stopped beating. N. Engl. J. Med..

[25] Barlow A.D., Metcalfe M.S., Johari Y., Elwell R., Veitch P.S., Nicholson M.L. (2009). Case-matched comparison of long-term results of non-heart beating and heart-beating donor renal transplants. Br. J. Surg..

[26] Mirshekar-Syahkal B., Summers D., Bradbury L.L., Aly M., Bardsley V., Berry M., Norris J.M., Torpey N., Clatworthy M.R., Bradley J.A. (2017). Local expansion of donation after circulatory death kidney transplant activity improves waitlisted outcomes and addresses inequities of access to transplantation. Am. J. Transplant..

[27] Shui K., Zhang H., Li T., Hou J., Lan G., Peng F., Yu S., Xie X., Dai H., Peng L. (2025). Clinical outcomes of kidney transplantation from expanded-criteria donors and KDPI>85% kidneys in deceased Chinese donors. BMC Nephrol..

